# Digitalising the past decades: automated ICD-10 coding of unstructured free text dermatological diagnoses

**DOI:** 10.1186/s12913-024-11761-y

**Published:** 2024-10-29

**Authors:** Sebastian Sitaru, Fabian Nhan, Christine Gasteiger, Daniel Rueckert, Tilo Biedermann, Alexander Zink

**Affiliations:** 1 School of Medicine and Health, Department of Dermatology and Allergy, Technical University of Munich, Biedersteiner Str. 29, Munich, 80802 Germany; 2 School of Medicine and Health, Institute of AI and Informatics in Medicine, Technical University of Munich, Munich, Germany; 3 Biomedical Image Analysis Group, Department of Computing, Imperial College London, London, UK

**Keywords:** ICD, Coding, Unstructured, Algorithm, Rule-based

## Abstract

**Background:**

Current digital medical databases record systematically coded diagnoses, but many legacy databases are full of hand-written, free text diagnoses, which can only be meaningfully analysed after mapping them to a coding system. While diagnoses can be extracted from full medical notes with good accuracy, no algorithm using only an unstructured free text diagnosis with no additional data has been published to date.

**Objectives/methods:**

Therefore, we sought to create an algorithm which maps hand-written German diagnoses from our clinical photography database to ICD-10 diagnosis codes, validate its output manually by dermatologists and analyse diagnosis counts over time as a proof-of-concept of its application.

**Results:**

Our rule-based algorithm mapped 50,884 unprocessed hand-written German free-text diagnoses covering five decades to ICD-10 codes, while reaching an accuracy of 82% against 817 dermatologist-validated diagnoses. Out of 41,021 data points with the highest algorithm confidence the top 3 identified diagnosis classes were psoriasis, eczema, and non-melanoma skin cancer. The number of ICD-10 codes belonging to chronic inflammatory diseases showed a seasonal pattern with peaks in July, and when analysed aggregated by year, peaks correlated to events such as new therapy classes for these diseases.

**Conclusion:**

Using the presented algorithm, it is possible to reliably match hand-written free text of German dermatological diagnoses to ICD-10 codes, thus enabling systematic analysis of legacy databases, making past medical knowledge accessible to today’s patient care.

**Supplementary Information:**

The online version contains supplementary material available at 10.1186/s12913-024-11761-y.

## Background

Digitalization is arguably one of the most important challenges in health care in the 21st century. One important aspect for clinical practice, research, statistics and billing alike is the coding of diseases to systems like the international classification of diseases (ICD) [1]. Currently, the most widely used version is the 10th edition (ICD-10) [2]. It has numerous adaptations and translations to multiple languages including German (ICD-10-GM) and is organized in 22 chapters consisting of multiple disease blocks each (in the form of XNN, where X is a letter and N is a number from 0 to 9) [3].

However, medical records predate these coding systems and their adoption by many decades and most historic medical data is only available hand-written making it unavailable for recent digital and AI developments and analysis. Making these older records available for systematic studies and teaching could provide significant value, since many skin diseases, especially dermatological diseases like later-stage syphilis, have historically decreased in prevalence to the point where they exist almost solely in textbooks in some parts of the world [4]. Same accounts for very rare skin diseases which are only seen every few years even in large skin departments such as Degos disease and therefore missed in digital analysis. Large-scale studies usually use prospective data or routine data obtained since widespread adoption of digital processes. From our clinical experience, this can lead to less circulating knowledge about these diseases, and thus possibly a decreased the quality of care for these patients. Furthermore, these data are not accessible to novel algorithms such as machine learning-based algorithms: Many diseases have been correlated with different factors using these technologies, using e.g., publicly available web-search and social media data as sources [5–7]. For skin diseases, patterns ranging from well-known seasonal patterns of e.g., atopic dermatitis with flare-ups in the winter [8] to lesser-known ones, e.g., toxicity in melanoma immunotherapy [9] have been identified.

In fact, in literature, this problem of coding free-text medical information has already been addressed in many studies. Several studies present algorithms to code cases based on hand-written medical notes with good accuracy [10–13]. Using newer artificial intelligence methods, e.g., artificial neural networks, even higher accuracies are possible [14–16]. Free-text pathological reports could also be coded to related ICD-10 diagnoses with good accuracy [17]. However, all these studies used the full text of medical notes or findings, whereas in some cases such as in this respective study, only a single hand-written free text diagnosis as source for the electronic record is available. To our knowledge, no algorithm converting the limited diagnosis free text to a systematic coding system has been described before.

Therefore, the aim of this study was to build and validate an algorithm which can assign ICD-10-GM codes to free-text diagnoses, while providing a proof-of-concept of the application of this algorithm, analysing the variation and correlation of the counts dermatological diagnoses over time, proving that our algorithm can be used for backwards compatibility of previous, free text systems, and that it can thus make past knowledge available for current practice.

## Materials and methods

### Data sources

The German free-text diagnoses of patients photographed from 1979 to 2022 at the Department of Dermatology and Allergy of the University Hospital of the Technical University of Munich were considered for this study. For each photo session, the requesting physician fills out a hand-written form stating patient, diagnosis, and body parts to be photographed. The photographer then manually uploads this information along with the images into a digital database. The diagnoses are therefore present in an unstructured free-text (string) form, entered by the photographer based on the hand-written information on the original form. All records present in the database were considered; none were excluded.

### Algorithm design

For mapping these free-text diagnoses to an ICD-10-GM code, challenges include the plethora of synonyms, possible typographic errors and word order changes present in such data. Therefore, our algorithm written in Python relies on different sources of German synonyms as well as approximate (fuzzy) string matching to address these points. The source code is available at https://github.com/Combo1/AKK.

First, we extracted synonyms from publicly available sources such as the official ICD-10-GM classification [3], Alpha-ID-SE, an official ICD-10-GM synonym database [18], and Altmeyer’s Dermatological Encyclopedia [19], which were compiled into a table of synonyms for each ICD-10-GM code. Like fuzzy text matching, the token set ratio based on the Levenshtein distance was used [20]. This approach extracts a common substring from the original diagnosis string and each entry in the synonym table. It then calculates the ‘fuzz ratio’ between the common substring and the remaining portion of the original string without the common substring, between the common substring and the remaining portion of a synonym table entry without the common substring, and lastly between the remaining portion of the original string without the common substring and the remaining portion of the synonym table entry without the common substring. Among the three comparisons the highest fuzz ratio is then defined as the token set ratio. The Fuzz ratio returns a value from 0 to 100, depending on the number of edits necessary. This ratio however suffers from the problem that it prefers short strings if they match, e.g., “steroid acne” and “acne” have a 100 (maximum) fuzz ratio. To combat this problem, we weigh the fuzz ratio with the Sørensen-Dice coefficient [21, 22] for each entry in the synonym table. If the coefficient (normalized to 100) is 5 points higher than the fuzz ratio, the match is used instead. Finally, a score indicating the confidence based either on the fuzz ratio or the Sørensen-Dice coefficient normalized to 100 is produced, in addition to the likeliest matching ICD-10-GM code. Overall, the algorithm outputs the likeliest matching ICD-10-GM code and a score from 0 to 100 indicating the closeness of the match.

While the ICD-11 is the most recent ICD version, a final version of the German translation was not available at the time of writing, which is why we used the much more widespread 10th ICD version [3].

### Validation study

Two dermatologists manually and independently validated the algorithm output of 500 randomly selected diagnoses each, totalling 1000 validated diagnoses. To maximise the validity of this experiment by maximising the sample size, we opted to have only one annotation per diagnosis. For each diagnosis, the dermatologists annotated the classifiability as defined by the ability of mapping the free-text diagnosis to an ICD code (Boolean variable), match of algorithm ICD-10 code and free-text diagnosis (Boolean variable), corrected ICD code where applicable. Examples of non-classifiable values include an empty field, or text which does not include a diagnosis (e.g., “Folgeaufnahmen vom 05.07.07”, translating to “follow-up photos from July 5, 2007”), but not simple typos (e.g., “aop. Ekzem”, for “atop. Ekzem”, meaning atopic eczema).

### Correlation to seasonal and patient parameters

The correlation analysis included all diagnoses with a score of 99 or 100. The top 10 ICD-10 diagnosis blocks were identified by total absolute frequency. The count of each diagnosis block was aggregated (as sum) by the month of the photo session. To investigate potential correlations to climate factors, data from the Climate Data Centre of the German weather service were used. For mean monthly temperature, data from 1980 to 2010 were used. For mean monthly precipitation, data from 1920 to 2022 were used. This corresponds to the full dataset publicly available from these data sources. Since our goal was to establish a correlation to seasonal patterns, which have been consistent for the past decades, we pooled all available data and the time periods are different to the ones in our dataset [23].

### Statistical analysis

Statistical analysis was performed using R v4.2.1. Data is presented as mean ± standard deviation if not otherwise specified. Statistical p values obtained by Student’s t test, R function *t.test*, if not otherwise specified.

### Data access, cleaning and linkage

The investigators had full access to the database population used to create the study population. No data cleaning and/or linkage to other data sources was performed.

### Data protection

According to local legislation (Bavarian hospital law/BayKrG, article 27, paragraph 4) it is permitted to use routine patient data for research purposes. Our data was furthermore processed in a completely anonymized manner. Therefore, no IRB approval and informed consent to participate was required for this study.

## Results

### Dataset description

We analysed 50,884 unprocessed, unstructured, free text German diagnosis of 27,301 individual patients with clinical images taken at our department from 1979 to 2022. Of these, 1000 diagnoses in total were manually validated, 59 (5.9%) were marked as non-classifiable because the free text was either not documented or could not be mapped to an ICD-10-GM diagnosis. Examples of correctly and incorrectly matched diagnoses, their scores and validation result are shown in Table 1.

**Table 1:** Examples of correctly and incorrectly identified text diagnoses

Free-text diagnosis	Matched ICD code	Algorithm score	Correct	Classifiable?
Pityriasis rosea	L42	100	yes	yes
LE-artiges Arzneimittelexanthem auf ein neues Zytostatikum	L27.0	100	yes	yes
Poyod. gangraenosum	L88	81	yes	yes
V. a. Hautmetastasen bei met. Mamma-Ca	C79.2	75.29	yes	yes
Erosives Granulationsgewebe	L92.9	89	yes	yes
M. Dupuytren bds.	M72.0	77	yes	yes
Arzneimittel E. auf Allopurinol	Z04.8	75	no	yes
bullöses Eysipel	J43.9	77	no	yes
Verruca seborrhoica	B07	100	no	yes
Atop. Ekzem	L30.9	100	no	yes
Hypereosinophile Syndrome	D59.5	100	no	yes
Pricktest, saisonale	L57.4	87.17	no	no

### Real-world algorithm performance

The study algorithm identified 50,884 diagnoses from 1137 ICD-10-GM blocks with a mean output score of 96.07 ± 9.37 (range 0-100) across the whole dataset.

**Fig. 1:**
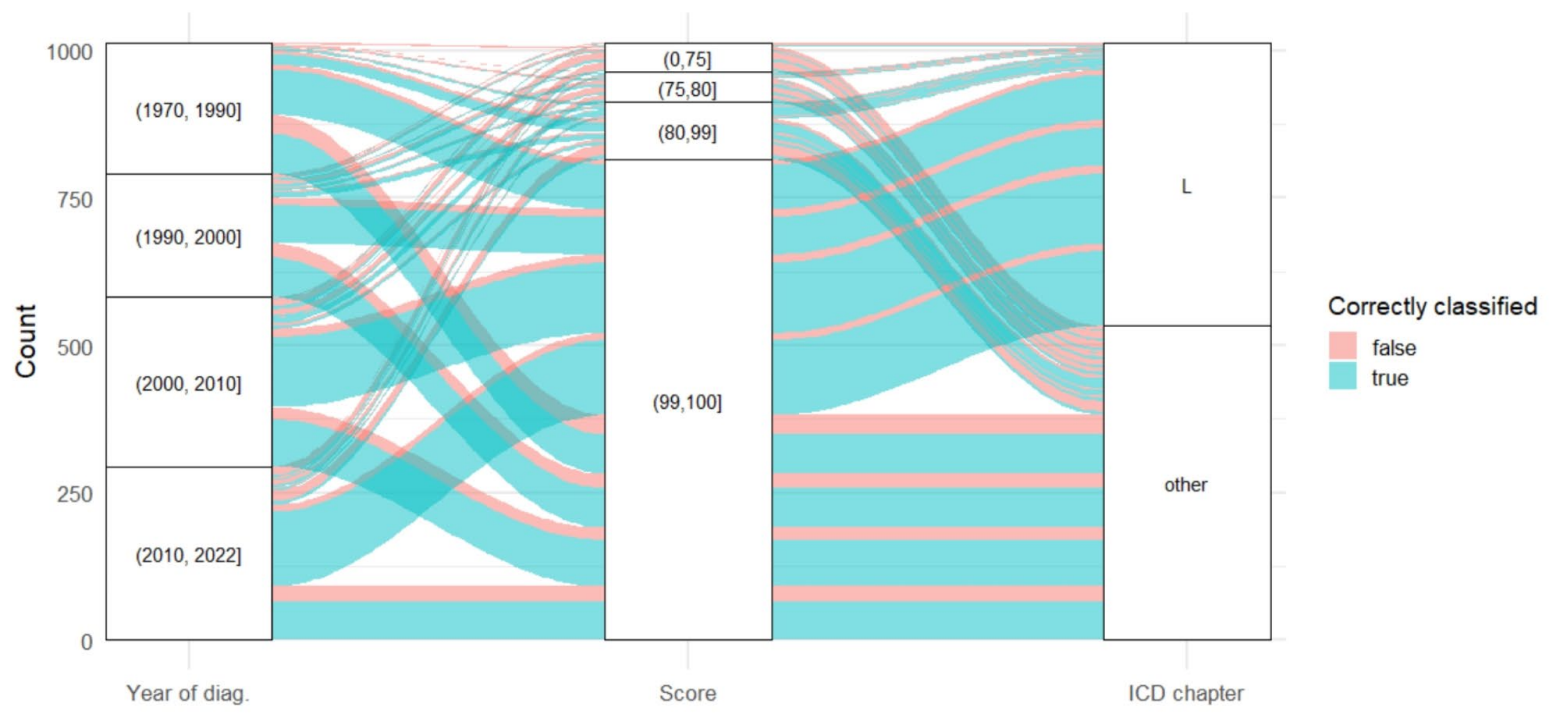
Algorithm performance by year of diagnosis, algorithm score, and ICD chapter. The alluvial plot shows the distribution and relationship of the year of diagnosis, algorithm score, ICD chapter, and the correct classification of the 1000 manually validated diagnoses

Based on the manual validation of 1000 diagnoses, the algorithm reached an accuracy of 75.5% (755/1000) with a mean output score of 95.92 ± 9.75 (Fig. 1). When considering only scores of 99 or 100, algorithm performance improves to 81.5% (666 of 817 diagnoses correct) with only one diagnosis being non-classifiable. The diagnoses are distributed evenly across the analysed years, and there is no significant relationship between the recorded year of diagnosis and correct classification (*p* = 0.7 by Wilcoxon’s rank sum test). The score distribution is also shown in Fig. 1; most classified diagnosis got a score of either 99 or 100 (816, 81.6%), and there is a significant relationship between the score output and correct classification (*p* < 0.01 by Wilcoxon’s rank sum test). The algorithm output score was significantly lower for the non-classifiable diagnoses than for the classifiable diagnoses (85.1 ± 17.34 vs. 96.6 ± 8.7, *p* < 0.05). Examples of correctly and incorrectly classified diagnoses are listed in Table 1. Of note, the free text “atop. Ekzem” (atopic eczema) was incorrectly classified as ICD-10 code L30.9, while the correct code would have been L20.0.

Other cases where the algorithm failed, listed in Table 1, include:

- “Arzneimittel E. auf Allopurinol”: Here, the most important part of the diagnosis, the exanthema, is abbreviated (“E”). Correct diagnosis: allopurinol-induced exanthematous drug eruption (L27.0).
- “bullöses Eysipel”: There is a typo, together with an additional adjective (corrected: “bullöses Erysipel”, bullous erysipelas). Of note, the typo-containing diagnosis text “Poyod. gangraenosum” (Table 1, upper part) was correctly identified as pyoderma gangrenosum.
- “Verruca seborrhoica“: An older term for a diagnosis nowadays called seborrheic keratosis (German: “Seborrhoische Keratose”).

### Time course of common dermatological diagnoses

**Fig. 2:**
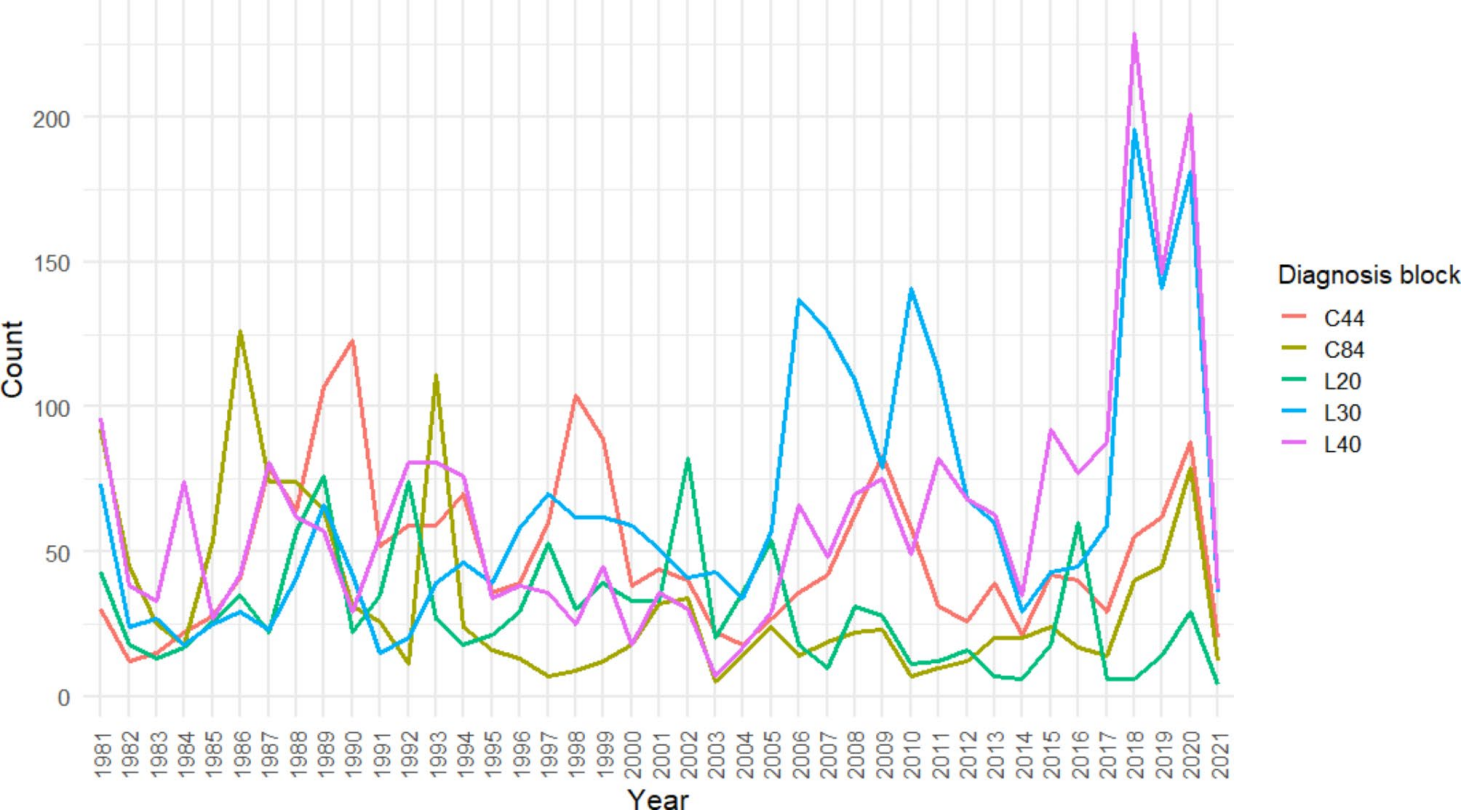
Counts of recorded diagnoses in the photo database by year. Count of the top 5 ICD-10 diagnosis blocks out of 41,021 matched diagnoses with algorithm score 99 or 100, aggregated by year of the photo session by sum

For a proof-of-concept application of our algorithm, a total of 41,021 diagnoses with a score of 99 or 100 from 1981 until 2021 were considered. The top 5 ICD diagnosis blocks by frequency were identified as L30 (dermatitis; 2626 counts), L40 (psoriasis; 2573 counts), C44 (other malignant neoplasm of skin such as for example basal cell carcinoma; 2013 counts), C84 (mature T/NK-cell lymphomas; 1341 counts) and L20 (atopic dermatitis; 1189 counts). Their count was aggregated by year for analysis (Fig. 2). Before 2006, the dominating photographed diagnoses were “mature T/NK cell lymphomas” and “other malignant neoplasms of the skin”, while for the rest of the analysed period, psoriasis and dermatitis were the most recorded diagnoses (Fig. 2). For dermatitis, prominent peaks were identified in 2006, 2010, 2018 and 2020. For psoriasis, prominent peaks are in 2018 and 2020.

### Seasonal variance of common dermatological diagnoses

**Fig. 3:**
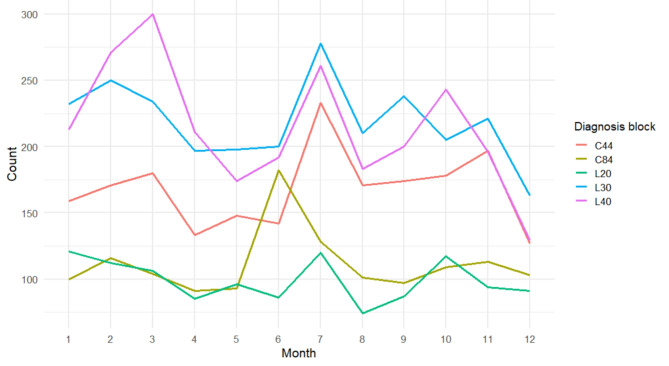
Seasonal variations in recorded diagnoses of the photo database. Count of the top 5 ICD-10 diagnosis blocks out of 41,021 diagnoses with algorithm score 99 or 100 over a year, aggregated by month by sum

The frequency of the top 5 ICD diagnosis blocks in our photo database identified earlier was furthermore analysed aggregated by month (Fig. 3). L30 and L40 are the most commonly occurring diagnosis blocks and for the most part, they follow a similar pattern with one peak in July, and smaller peaks in the beginning of the year (psoriasis: March, dermatitis: February). The highest value of the psoriasis diagnosis count is in March, the lowest is in December, while the highest value of the dermatitis diagnosis count is in July, and the lowest is in December. A statistically significant month-wise correlation between the count of overall photo sessions and mean monthly temperatures or mean monthly precipitation in Germany could not be found (Supplementary Figs. 1 and 2; Spearman’s rho = -0.014, *p* = 0.97, and rho = -0.22, *p* = 0.49, respectively). Also, Spearman correlation analyses of month-wise counts of the top 5 diagnosis blocks and mean monthly temperatures or mean monthly precipitation in Germany yielded statistically non-significant results.

## Discussion

The aim of the study was to create and validate an algorithm for the classification of German free-text diagnoses to an ICD-10 code and test its use by analysing the time course of the counts of recorded diagnoses. Our rule-based algorithm mapped 50,884 raw German free-text diagnoses of 27,301 patients from 1979 to 2022 to a corresponding ICD code, with an accuracy of 81.5% in a subset of 817 manually validated diagnoses with an algorithm output score of 99 or 100. The analysis of the diagnoses in our database revealed the top 3 photographed conditions by ICD blocks being psoriasis (L40), dermatitis (L30), and non-melanoma skin cancer (C44). From the 1980’s to the early 2000’s, mostly C44 and C84 (T/NK cell lymphomas) were photographed, while thereafter psoriasis and dermatitis were the dominant diagnosis. Albeit a seasonality in the month-wise number of recorded diagnoses could be established, no correlation could be established with either mean local monthly temperature or precipitation.

The task of classifying medical texts to ICD diagnoses has already been attempted numerous times before, however only from larger corpuses of text, like e.g., histopathological reports or medical notes [14–16]. Even though direct comparison is difficult because of different input data, these algorithms showed comparable trends in performance as ours, the latest published one for example reaching an F1 score of 0.715 [14]. Compared to the previous approaches however, we used uncommon and domain-specific additional data sources such as the Alpha ID SE, which is an ICD-10-GM synonym list, and Altmeyer’s encyclopaedia, which is a dermatological online encyclopaedia, to compile a synonym list [18, 19]. Therefore, our approach, which is novel for this specific task, seems to offer similar, state-of-the-art performance. Furthermore, the output score was significantly lower for manually identified non-classifiable diagnoses than for classifiable diagnoses, meaning that the output score has a predictive value on the accuracy of the classification.

Crucially, our algorithm enables the large-scale analysis of disease patterns from limited unstructured free-text diagnoses, which is the technically easiest method of recording diagnoses and has been used in many systems including our photo database, especially in the past. To provide a proof-of-concept of the application of this algorithm, we first analysed the obtained ICD-10 blocks by year, which revealed completely different patterns before and after about 2006: Before 2006, mostly malignant skin diseases (C44 and C84) were photographed, while in 2006 and 2010, eczema (L30) diagnoses peaked. Because of limitations of our algorithm explained below, the block L30 also includes most of the atopic eczema diagnoses. Usually, clinical photos are taken for general documentation purposes, for very rare or unusual conditions, and for evaluating therapeutic response, especially for new therapies. Therefore, the time course could be interpreted as follows: The 2006 peak could be explained due to experimental therapy of (atopic) dermatitis with omalizumab, which was received market authorisation for chronic urticaria in 2005 in Germany and was subsequently used in some clinical trials and as an individual experimental therapy [24]. However, the therapeutic effects were not consistent, so this therapy was not pursued further after initial trials, at least in our department [25]. In 2010, trials were conducted with a combination of immunoadsorption and omalizumab, for which patients were regularly photographed for a total of ~ 50 photo sessions, which can explain this peak [26]. The 2018 peak of dermatitis diagnosis counts coincides with a milestone in atopic dermatitis therapy: European market authorization for dupilumab. The build-up and peak of psoriasis diagnosis counts in 2018 can also be explained by the market authorization of an unprecedented number of targeted psoriasis therapies: in 2017, tofacitinib, guselkumab, brodalumab, and in 2018, certolizumab pegol, received EU market authorization. 2020 marks another peak in dermatitis and psoriasis diagnosis counts, which could be explained by EU market authorization of barictinib for atopic dermatitis, and risankizumab for psoriasis one year prior. Besides market authorisations of new drugs however, organisational changes at our department such as personnel changes or changes within the structure of the health system, e.g., reimbursements for certain diagnoses, can trigger changes in the population seeking care at our department and thereby affect the frequency of diagnoses.

When grouping diagnosis counts by month, we could observe peaks for most of the top 5 disease codes, including psoriasis, dermatitis, and non-melanoma skin cancer, in the summer and spring months of the northern hemisphere (March and July). A month-wise statistical correlation to mean temperature or mean precipitation could not be established. For atopic dermatitis, web-search data in Europe revealed similar trends with peaks of search volume, and thus disease burden, in the beginning of the year (January) and later in the year (March) [27]. The pattern for psoriasis also fits with previous reports, where a similar time course in the initiation of biologic and non-biologic therapies, corresponding to high case load and/or disease burden, were found, with peaks in spring and summer [28]. Indeed, four of the top 5 analysed diagnoses spiked in July. A possible explanation could be that patients are not dressed in as many layers in the summer months, and thus the threshold to have photos taken is lower, as opposed to winter. Beyond dermatology, the seasonality of patient visits for many conditions including allergic asthma and mental health disorders have been established, providing potential future applications of the presented algorithm [5, 29, 30].

In the age of artificial intelligence and machine learning, we opted for a rule-based approach, which might not be as accurate as newer machine learning approaches such as artificial neural network-based natural language processing [14], however rule-based approaches have the benefit of built-in explainability and therefore offer easier debugging and extension possibilities without the need for extra tools or methods [31]. Crucially, our algorithm is much more flexible than machine learning approaches, as only the synonym table would need to be updated for adoption for other coding systems or languages. By contrast, most machine learning approaches require generation of new data, and then training, which arguably is more resource intensive [14].

### Limitations

Despite its overall good performance, the algorithm also suffers some limitations: Clinicians often use abbreviations instead of the correct medical term for diagnosis, causing false grouping of diagnoses. For example, “atop. Ekzem” (short for atopic eczema) gets classified as unspecified eczema (L30.9), even though the correct ICD-10 code would be L20.0, which must be considered when interpreting the data.

The algorithm also incorrectly classified diagnoses with abbreviations obscuring the actual diagnosis (“Arzneimittel E. auf Allopurinol”, correct diagnosis: allopurinol-induced exanthematous drug eruption), because it is not context-aware. Making the algorithm context-aware could be achieved by including more advanced natural language processing techniques like embeddings. Specific typos in the diagnosis texts, when using additional qualifiers (“bullöses Eysipel”, correct diagnosis: bullous erysipelas), too, can lead to an incorrect diagnosis. Weighting the Levenshtein distance based on different character substitutions could alleviate this problem in future versions. In another case (“Verruca seborrhoica”), the algorithm failed to map the older term “verruca seborrhoica” (= seborrheic wart) to the currently used term “Seborrhoische Keratose” (= seborrheic keratosis). Due to its practical relevance, our algorithm classifies to the ICD-10(-GM) system, which was published in 1994 [3], while our free-text diagnoses date back to 1979. Since diagnosis terms and semantics change over time, we used a synonym table based on the dermatological Altmeyer’s encyclopaedia amongst others, which includes both current and historical synonyms. A more specific analysis of the classifiability of diagnoses, focusing on the differences of diagnosis names over time, could therefore help to further improve the performance of the algorithm.

Also, our data source, i.e., the photo database, suffers from selection bias, since photographs are routinely taken for documentation of unusual or rare skin findings or follow-up of chronic diseases being treated with new therapies, but not necessarily for common, easy-to-diagnose skin diseases. Furthermore, our department preferentially sees complex cases which have not been solved by other health care providers, e.g., primary care physicians or dermatologists, before. Therefore, the diagnoses counts should be interpreted carefully, and might not be reflective of actual disease burden or prevalence.

### Conclusion and outlook

Overall, our rule-based algorithm can classify German dermatological free-text diagnoses to ICD-10 codes with good accuracy, thus empowering systematic analysis of databases using legacy, free-text coding systems, and subsequent correlations to environmental and other factors. The approach shown here can very easily be extended and adapted to any language and specialty and can therefore help to make available past knowledge for current and future medical care across all fields.

## Supplementary Information

Supplementary Material 1.

## Data Availability

The algorithm code is available at https://github.com/Combo1/AKK. The raw data are not published due to privacy concerns, but are available upon reasonable request from the corresponding author.
