# Supplementary material for: Digitalising the past decades: automated ICD-10 coding of unstructured free text dermatological diagnoses
Source: BMC Health Serv Res. 2024 Oct 29;24:1297. doi: 10.1186/s12913-024-11761-y (PMC11520645; doi:10.1186/s12913-024-11761-y)
Supplement: Supplementary file 1 — Supplementary Material 1. [file 12913_2024_11761_MOESM1_ESM.docx]

**Supplementary material for: Digitalizing the past decades: Automated ICD-10 Coding of Unstructured Free Text Dermatological Diagnoses (Sitaru *et al*)**


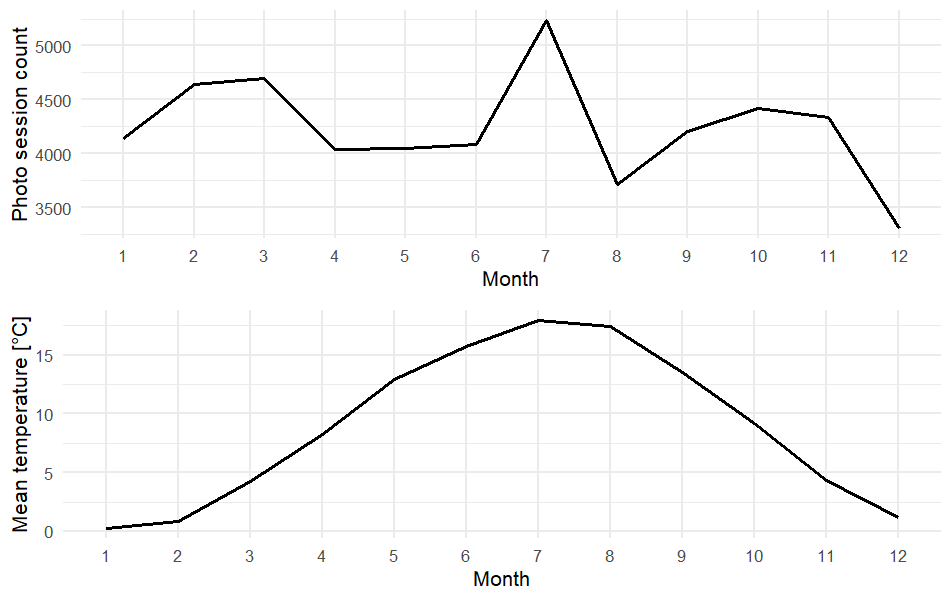


**Supplementary Figure 1.** Total photo session count vs. mean temperature per month as a visualisation of the correlation between these two variables. Spearman’s rho = -0.014, p = 0.97.


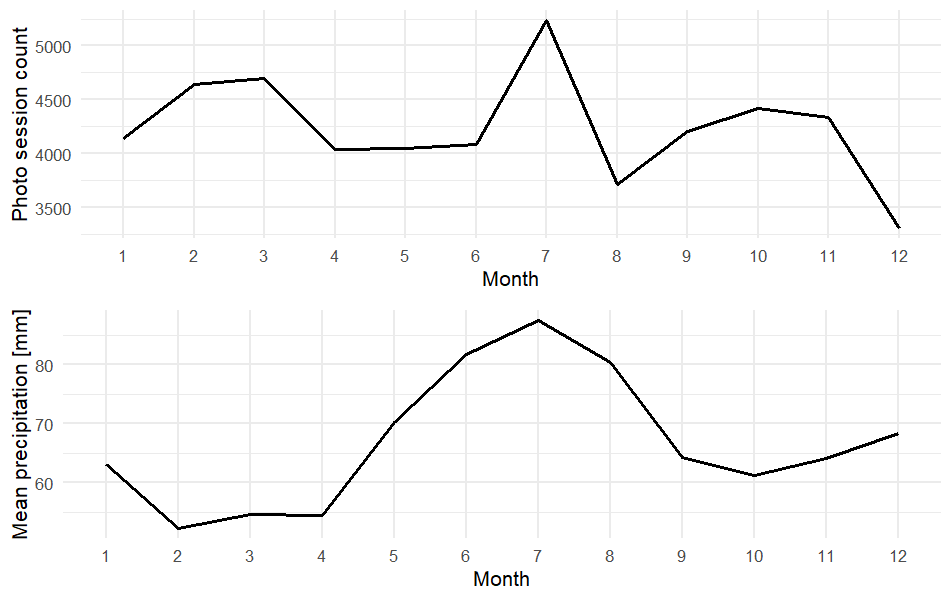


**Supplementary Figure 2.** Total photo session count vs. mean precipitation per month as a visualisation of the correlation between these two variables. Spearman’s rho = -0.22, p =0.49.
